# Beyond Synchrony: Joint Action in a Complex Production Task Reveals Beneficial Effects of Decreased Interpersonal Synchrony

**DOI:** 10.1371/journal.pone.0168306

**Published:** 2016-12-20

**Authors:** Sebastian Wallot, Panagiotis Mitkidis, John J. McGraw, Andreas Roepstorff

**Affiliations:** 1 Max Planck Institute for Empirical Aesthetics, Frankfurt, Germany; 2 Interacting Minds Centre, Aarhus University, Aarhus C, Denmark; 3 Interdisciplinary Center for Organizational Architecture, Aarhus University, Aarhus C, Denmark; 4 Center for Advanced Hindsight, Duke University, Durham, North Carolina, United States of America; 5 Departments of Religious Studies and Central American Studies, California State University, Northridge, Northridge, California, United States of America; University of California Merced, UNITED STATES

## Abstract

A variety of joint action studies show that people tend to fall into synchronous behavior with others participating in the same task, and that such synchronization is beneficial, leading to greater rapport, satisfaction, and performance. It has been noted that many of these task environments require simple interactions that involve little planning of action coordination toward a shared goal. The present study utilized a complex joint construction task in which dyads were instructed to build model cars while their hand movements and heart rates were measured. Participants built these models under varying conditions, delimiting how freely they could divide labor during a build session. While hand movement synchrony was sensitive to the different tasks and outcomes, the heart rate measure did not show any effects of interpersonal synchrony. Results for hand movements show that the more participants were constrained by a particular building strategy, the greater their behavioral synchrony. Within the different conditions, the degree of synchrony was predictive of subjective satisfaction and objective product outcomes. However, in contrast to many previous findings, synchrony was negatively associated with superior products, and, depending on the constraints on the interaction, positively or negatively correlated with higher subjective satisfaction. These results show that the task context critically shapes the role of synchronization during joint action, and that in more complex tasks, not synchronization of behavior, but rather complementary types of behavior may be associated with superior task outcomes.

## Introduction

Research on joint action has shown that synchronization between individuals is associated with better rapport and performance [1–4]. Synchrony in neural and physiological activity has been hypothesized to be a mechanism for empathy, mutual understanding, and trust [5–7]. Multiple notions and measures of synchrony have been used in the literature on interpersonal coordination, but the common denominator tends to be that synchrony implies correlation between two or more (behavioral or physiological) signals when those signals are temporally aligned (cf. [6,8,9]). Hence, we use temporal correlation between aligned signals (i.e., at lag 0) as our operational definition for synchrony in the current paper.

Most studies that have shown beneficial effects of synchrony relied on very simple tasks that highly constrained participants’ behavioral options, or on tasks in which behavioral synchrony was an explicit goal of the joint action—such as swinging pendulums together [10], rocking together in rocking chairs [11], or clapping in a movie theater [12]. Synchrony has also been observed in more naturalistic settings, such as in sharing of postures between individuals in a classroom setting [13], between members of opposite-sex-dyads that engage in conversation and displayed courtship-like behavior [14], or between movements of a therapist and client in a therapeutical setting [15]. Here, measures of synchrony were generally positively associated with liking and rapport between individuals.

Besides synchrony, recent research reports have shown that another basic form of coordination is important for interpersonal interaction, namely complementarity [16]. For example, dyads were asked to move dots on a screen together, so that one participant had to move a dot back and forth from the lower-left to the upper-right and the other one from the lower-right to the upper-left [17]. However, when the dots crashed into each other during these oscillatory movements, the trial was counted as a failure. Under such circumstances, participants do not adopt straight synchronous behavior (e.g., producing in-phase oscillatory arm movements), but exhibit ‘complementary’ task dynamics: while one would produce oscillatory movements in a straight line, the other would produce movements in broad circles. Together, these ‘complementary’ behaviors were more functional in order to minimize collision than synchronized behavior.

Another instance of what has been dubbed ‘complementary’ is turn-taking dynamics during conversation, where participants do not talk and pause at the same time (or synchronize in terms of other speech features, such as prosody), but perform different speech actions at the same time [18]. However, complementary actions are not yet coherently defined. For example, movement patterns in the dot-movement task are quantitatively similar, but not time-locked at lag0 (i.e., exhibiting the same movement trajectories at the same time), while speech actions in the conversation task are qualitatively different, but in many ways near-synchronous (e.g., one participant talks while the other one is silent and vise-versa).

Even though a more strict definition of ‘complementary’ coordination has yet to be provided [16,19], the recent interest in complementary forms of interpersonal interaction hints at the fact that researchers are increasingly interested in forms of social coordination that are beyond synchrony. Although we have discussed some exceptions above [13–15], it is noteworthy that the majority of studies that have demonstrated positive effects of synchrony were not outcome-oriented, but rather focused on interpersonal coordination as an end in itself [20,21]. When participating in more naturalistic tasks, people encounter considerably more coordination options permitting multiple means for achieving similar ends. Accordingly, researchers have called for the investigation of more complex tasks, where joint action requires planning, and is directed towards the solution of problems, in order to broaden the scope of joint action research [22].

### Design and hypotheses

In the present investigation, we aim at investigating the role of synchronous coordination dynamics in a relatively open, naturalistic joint action task. In particular, we hypothesize that in the presence of multifinality (i.e., when many potentially useful ways to archive a task outcome are present) diversification of action is likely to occur, and complementary forms of interaction will in many cases supersede synchronous kinds of interaction. In contrast, as the degrees-of-freedom of how to go about a task are constrained, synchronous kinds of interaction are more likely to occur (such as in the case where coordination of action is an implicit or explicit goal in itself). Specifically, this implies that the degree of synchrony in setups that allow for flexible action-coordination among participants should drop significantly compared to conditions where participants’ flexibility of action-coordination is reduced. In how far changes in the degree of synchrony are associated with increases or decreases of subjective and objective task outcomes (satisfaction, product quality) is an open question.

The present study tests this hypothesis by varying degrees of freedom in the interactions by manipulating the role of participants in a dyadic joint action task with an outcome-oriented component. In the current study participants were asked to build beautiful and efficient model cars together. Dyads had the greatest freedom in how to structure their interaction toward the goal of building the model car in the so-called “egalitarian cooperation” condition (EC): where they received no particular instructions on how to divide work during the task, but were simply required to work together. In contrast, participants in the “hierarchical cooperation” condition (HC) were assigned different roles: one participant had to make all design decisions while the other could only assist in the building process upon request. This reduced the freedom of interpersonal coordination participants were not freely allowed to interaction anymore, and only certain interaction patterns were permissible thus making the coordination process itself a goal of the interaction. Finally, we ran a control condition—the “turn-taking” condition (TT)–where participants received explicit instructions to divide the work by taking turns building the car, so that only one participant would actively build while the other had to wait for the next turn. This severely limited the forms of coordination that participants could employ, and effectively eliminated online interaction during the building process. It should be noted that participants performed HC twice, in back-to-back sessions, in order to give each participant the chance to assume the role of the leading car designer. That means that while we employed three different kinds of building conditions in terms of instructions (i.e., TT, HC, and EC), we recorded four building sessions from each dyad (i.e., 1xTT, 2xHC and 1xEC).

The three building conditions exhibit important variations of how people coordinate their actions: TT highly constrained the interaction between participants, providing few possibilities for online interaction; HC afforded more interaction opportunities, but the goal was not only to coordinate joint actions towards the building process, but also towards each other, increasing the focus on the interaction process itself; finally, EC permitted the greatest flexibility for participants’ coordination towards the shared goal.

Participants’ hand movement accelerations and heart rates were recorded. Synchrony within hand movement accelerations, as well as synchrony within heart rate profiles was calculated using Multi-Dimensional Recurrence Quantification Analysis (from here on MdRQA). Heart rates were measured as potential markers of shared emotional states [6] that might influence group performance [23] and mutual trust [7].

This allowed us to investigate whether measures of synchrony are affected by different constrains on the interaction process and to investigate whether synchrony had a beneficial role in complex joint action task, where participants’ actions are oriented towards a shared goal, rather than towards the behavior coordination process itself. To that end, we examined how behavioral and physiological synchrony related to subjective and objective outcome measures (functionality and aesthetics). Specifically, we investigated: 1) whether process measures of the interaction (i.e., synchrony in hand movement activity and synchrony in heart rate) were predictive of subjective (i.e., self-reports capturing participants’ experienced fun, task difficulty, and satisfaction) and objective outcomes of the interaction (i.e., product measures regarding car performance and aesthetic appeal of the cars; and 2) whether the constraints of the specific conditions (EC, HC, TT) shaped subjective and/or objective outcomes.

This allowed us to examine whether in a relatively open and unconstrained task (building a model car together) would be characterized by positive effects of synchrony (e.g., positive correlations between synchrony and subjective satisfaction as observed previously using simpler task designs) or by a different kind of relationship (e.g., negative correlations between synchrony and subjective satisfaction).

## Method

### Participants

Seventy-four students from Aarhus University participated in the experiment (average age: 23.5yrs, *SD* = 3.5yrs) and were randomly assigned to pairs. Half of the participants (i.e., 37) were female; the other half were male. Of the resulting 37 dyads, 13 were male-only dyads, 13 were female-only dyads, and 11 were mixed male/female dyads. All participants reported that they did not know their partner beforehand. Using standardized forms, the pairs were instructed to build LEGO cars. Participants were compensated with 350 DKK (≈ 47 EUR). The protocol was reviewed and approved by the Ethics Committee for Region Midtjylland, Denmark. All participants provided written consent.

### Procedure

**Fig 1:**
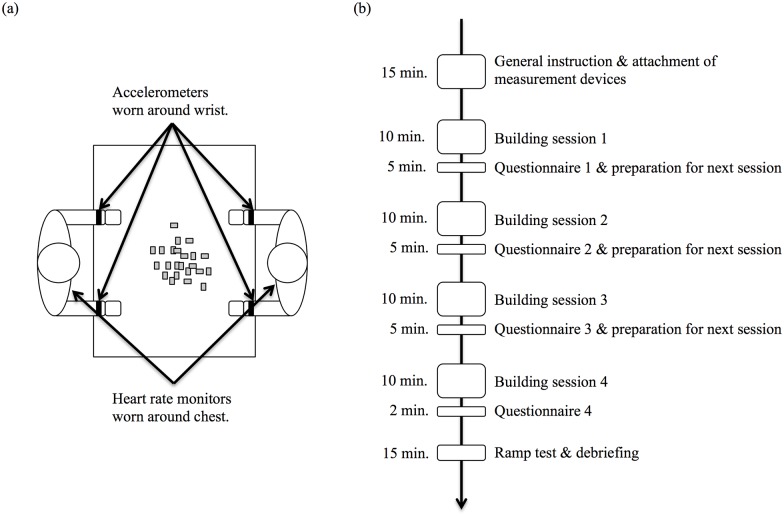
Overview of the experimental setup and time-course. (a) Setting in which participants built the cars and the location of the measurement devices. (b) Time-course of the experiment.

The order of building conditions was randomized between dyads, and a full experimental session lasted 70–90 minutes. During the experimental set up, heart rate monitors and hand accelerometers were attached to the participants. They were brought into a quiet room and seated at a table opposite one another (Fig 1a) and were instructed to jointly build aesthetically pleasing and functional cars (by the term “functional” it was meant that the cars should travel as far as possible after successfully being rolled down a ramp that was shown to participants at the beginning of the experiment). Standardized instructions were presented to participants on a sheet of paper before the beginning of each building session. Thereafter, participants were provided with a set of LEGO bricks. The bricks were two combined sets to build cars (i.e., the Lego-Creator-Sets 5763 and 6913), providing participants with many generic bricks, but also a variety of specific car and car-like parts (wheels, axes, lights, as well as hood, grill, trunk and door parts), 289 pieces in total. Dyads could freely communicate—all that was required was that they remained seated during the building process (see Fig 1a for a schematic of the task setup). Each building session took 10 minutes (dyads were forewarned 2 minutes and 1 minute before the 10 minutes were up, at which point the model was taken away, even if it had not yet been fully completed). Apart from two instances in which dyads finished their model car early, participants always worked well into the last minute to finish the car. All dyad finished their cars in time, producing functional cars (except for one dyad, whose car did have wheels, but the wheels were too close to the chassis and did not move adequately).

Before each of the four building sessions, participants received instructions related to one of the three building conditions (EC, HC, TT): in EC, participants were simply directed to build a car together; in HC, participants were also building the car together, but one participant had to make all design decisions while the other could only assist (this building condition was run twice in back-to-back sessions, so that each participant played the role of designer or assistant); in TT, participants were instructed to build the car by taking turns—one participant would offer directions for the first task (e.g., construct four wheels and axes) and the other participant would implement this suggestion, following which the participants switched roles so that the one who had implemented the first suggestion would suggest the next task (e.g., attach the wheels to a chassis), and so forth. Upon completing each session, participants rated their cooperation in the building task. Objective product performance measures of the car were taken at the end of the experiment (Fig 1b).

#### Equipment and measures

Participants wore ActiGraph GT3X+ accelerometers [24] on each wrist and a Polar Team2 [25] heart rate monitor around the chest. The accelerometers sampled hand acceleration at 100Hz. The heart rate monitors recorded participants’ heartbeats as beats-per-minute (BPM) at 1-second intervals. Due to problems with the heart rate equipment, data was lost from eight participants during HC and from six participants during EC. Visual analogue scales were used to measure six self-report items (see the Supporting Information).

Regarding the questionnaire items ‘fun’, ‘cooperation’, ‘difficulty’, ‘effort’, and ‘product satisfaction’, responses were averaged across dyads for each item and session to a dyad score. For the item ‘control’, the absolute difference of the scores was calculated. Here, bigger differences indicate the perceived power asymmetry, while smaller differences indicate the perceived equality between participants (see S1 Table in the Supporting Information, for a summary of the correlation of participants’ answers within dyads).

To gauge performance, each car was assessed five times using a procedure that measured maximum distance traveled (cm) after release of the car from the top of a fixed ramp. Data from one car could not be collected, as it lacked moving wheels. To gauge aesthetics, standardized photos were taken of each car and assessed by 9 naïve raters on a scale from 1–5. Each rater rated each car individually, based on a frontal and lateral picture of the car. The order of presentation was randomized. A single aesthetic appeal score was calculated for each car by averaging across all 9 ratings. A correlation table displaying the correlations among the 9 raters can be found in the Supporting Information (S2 Table). Finally, the size of the car was measured by a count of its component bricks. Note, however, that bricks came in a variety of sized. Hence, the brick count does not strictly equal the volume or density of the car, but is rather a correlate of those quantities and reflects the productivity of the participants to add together many pieces in a fixed amount of time.

As the HC was recorded twice for each group, yielding double the amount of data points, all values were averaged across the two recordings to yield the same number of data points as in the other two conditions (see also the Preliminary Analysis section below). A correlation table displaying the correlations among the subjective and objective outcome measures can be found in the Supporting Information (S3 Table).

### Data analysis

Hand acceleration data were down-sampled to 10Hz, providing sufficient resolution for the observed level of activity. MdRQA was used to quantify synchrony of the activity measures and heart rate signals within each dyad [6,26]. We chose MdRQA over Cross-Recurrence-Quantification Analysis (CRQA)–which is recurrence-based technique that has been more widely used in the literature on coordination dynamics—because we needed to assess synchrony among up to 4 signals simultaneously (i.e., 4 hand movement signals from each dyad), which is not possible with CRQA. Besides the number of signals one can simultaneously analyze with each method, there are two other differences between the methods that are noteworthy: In CRQA, the resulting (cross-)recurrence plot is based on the distances *between* the two individually embedded signals in phase space. In MdRQA, multiple signals are embedded together, and the resulting recurrence plot is based on the distances *within* the multidimensional signal. This means, that the recurrence plot underlying MdRQA is, in contrast to CRQA, symmetrical about the main diagonal, and hence does not allow for an analysis of time-lagged behavior among the multiple constituent signals (for example, it could be of interest to see whether one participant is leading the building process or whether participants switch in who is leading and who is following; questions like these cannot be answered with MdRQA).

MdRQA was used to assess synchrony separately for hand-movements and heart rates (i.e., synchrony of hand movements was computed based on the four hand acceleration signals obtained from each dyad, and synchrony of heart rates was computed based on the two heart rate signals obtained from each dyad).

MdRQA is a time series analysis technique that measures the relationship between two *or more* time series and can be used to quantify their degree of synchrony [27]. MdRQA was inspired by work of Thomasson and colleagues [28], who were the first to use recurrence analysis of the Euclidean distance of a multivariate signal (EEG channels) to quantify the synchronization of multivariate brain activity. MdRQA [27] is an extension of cross-recurrence analysis, which is a nonlinear correlation analysis that quantifies the evolution of two time series [29]; it is a prominent technique for the analysis of temporal coordination and has been used in a variety of joint action studies [6,19,26,30,31]. To conduct recurrence analysis, the time series are projected into a phase space by the method of time-delayed embedding. The time series are plotted against themselves with a time lag, according to a delay-parameter (from here on, DEL). The number of times that the data are plotted against themselves is determined by the dimension-parameter (from here on, DIM). The time series are normalized before the embedding procedure to ensure that the recurrence measures are not overly influenced by the magnitudes of participants’ hand movement accelerations but, rather, are based on the sequence of accelerations [26].

**Fig 2:**
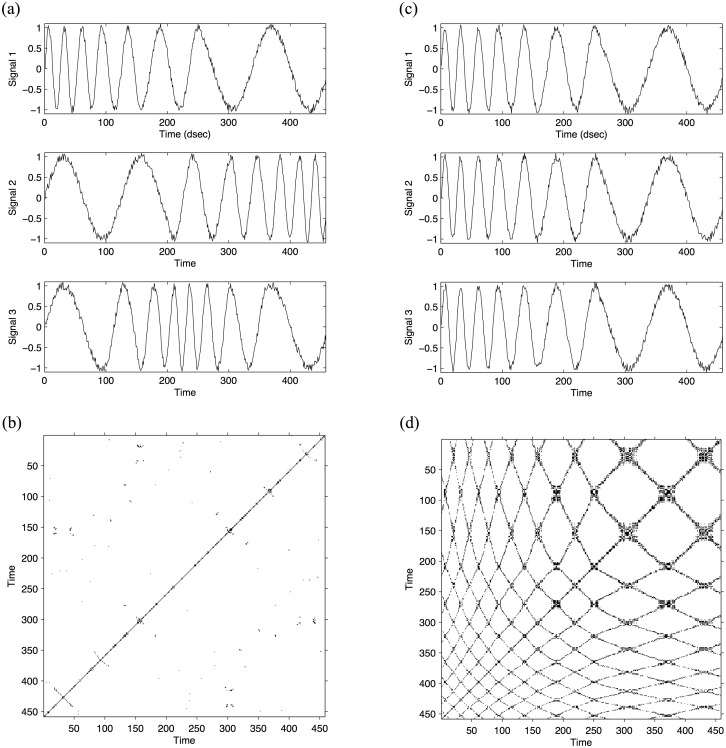
Illustration of MdRQA on artificial continuous signals, i.e., sine waves with added random noise. Fig 2a depicts a set of three sine waves that exhibit a relatively low degree of synchrony together with their associated recurrence plot (2b). Fig 2c depicts a set of three sine waves that exhibit a relatively high degree of synchrony with their associated recurrence plot (2d). The recurrence plot in 2b is sparsely populated compared to 2d. Furthermore, the recurrence points are much less connected in 2b compared to 2d, as can be quantified by %Determinism. %Determinism is defined as the number of points in a recurrence plot that form *adjacent diagonal line structures* divided by the number of all points on the plot: while the plot in 2b exhibits %Determinism = 18.6%, the plot in 2d exhibits %Determinism = 54.1%. This figure was adapted from [7] with permission from Elsevier and Copyright Clearance Center (license number: 3926230267291).

Fig 2 provides an example. Let us say we have two sets of three signals (*S*) each, all with the same average frequency content (*f*) plus random noise (*e*). *S* = sine(*f*) + *e*. However, *f* is changing over time. While the changes of *f* are different for the three signals in set 1 (Fig 2a), the changes of *f* are the same for the three signals in set 2 (Fig 2c). Hence, the two sets of signals differ in the degree of synchrony among them, where set 1 (Fig 2a) exhibits a relatively low degree of synchrony, while set two (Fig 2c) exhibits a relatively high degree of synchrony.

The evolution of these two sets of signals can now be represented as a recurrence plot. The recurrence plot represents each set of three signals as the distances between the data points. That is, if the three signals exhibit very different dynamics, the distances between the signals will be large. If the three signals exhibit very similar dynamics, these distances will be small. Small distances that fall below a pre-defined threshold yield recurrent structures on the recurrence plot, while large distances do not. Hence, in a recurrence plot, periods with a high degree of synchrony are marked by black areas (recurrence), while periods with a low degree of synchrony are marked by empty spaces (no recurrence). Fig 2b shows the associated recurrence plot for the set of signals with a low degree of synchrony, while Fig 2d shows the associated recurrence plot for the set of signals with a high degree of synchrony. As can be seen, the set of signals with a low degree of synchrony exhibits fewer recurrences (black areas), while the set of signals with a high degree of synchrony exhibits more recurrences.

**Fig 3:**
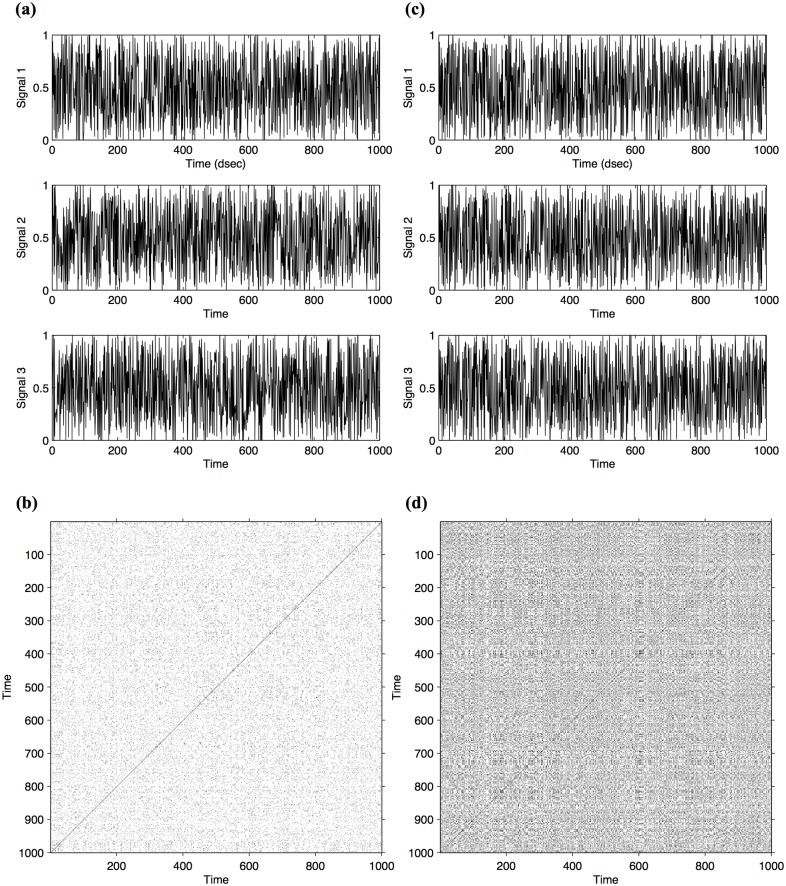
Illustration of MdRQA on artificial stochastic signals, i.e., random numbers drawn from a uniform distribution. Fig 3a depicts a set of three signals that exhibit a relatively low degree of synchrony together with their associated recurrence plot (2b). Fig 2c depicts a set of three signals that exhibit a relatively high degree of synchrony with their associated recurrence plot (2d). The recurrence plot in 2b is sparsely populated compared to 2d. Furthermore, the recurrence points are much less connected in 2b compared to 2d, which can again be quantified by the measure %Determinism, which is defined as the number of points in a recurrence plot that form adjacent diagonal line structures divided by the number of all points on the plot: while the plot in 2b exhibits %Determinism = 7.7%, the plot in 2d exhibits %Determinism = 33.6%.

As shown in the example provided in Fig 3, MdRQA can equally be applied to stochastic signals: all signals presented in Fig 3 are drawn from a uniformly distributed random process [0,1]. While the three signals in Fig 3a are three independent realizations of that process, the three signals in Fig 3c are all correlated with each other at lag0 by virtue of their relation to a fourth signal (*S4*), such that Signal 1 (*S1*) is defined as *S1*_*n*_ = *S1*_*n*_ + *wS4*_*n*_, Signal 2 (*S2*) is defined as *S2*_*n*_ = *S2*_*n*_ + *wS4*_*n*_, and Signal 3 (*S3*) is defined as *S3*_*n*_ = *S3*_*n*_ + *wS4*_*n*_, where *w* is a weighting parameter. Hence, the two sets of stochastic signals differ in the degree of synchrony (lag0 correlations) among them, where set 1 (Fig 3a) exhibits no synchrony, while set two (Fig 3c) exhibits a certain degree of synchrony, which is again evident from the density of recurrence observed in their respective recurrence plots (cf. Fig 3b with 3d).

To assess synchrony more thoroughly, however, it is important to determine whether the recurrences in the recurrence plots connect to each other. Isolated points of recurrence could simply be the result of chance—two independent random variables could exhibit many individual recurrences, if variables are drawn from the same distribution and that distribution has a restricted range of values. In contrast, we are interested in correlations between variables over time, which are displayed in the recurrence plots as recurrence that perseveres over time. That is to say, instances where the three signals do not merely cross each other accidentally, but exhibit continued coordination with each other. In recurrence quantification type of analyses, the extent to which coordination occurs in terms of such “trajectories” can be captured by the measure %Determinism [29,32]. %Determinism is defined as the number of points in a recurrence plot that form adjacent diagonal line structures divided by the number of all points on the plot and has been used in several studies to quantify interpersonal coordination [19,31]. Another measure that is applicable in this context is %Laminarity [29]. %Laminarity is defined as the number of points in a recurrence plot that form *adjacent vertical line structures* divided by the number of all points on the plot. However, both quantify temporal coordination somewhat differenty. While %Determinism captures how a signal is organized in terms similar sequences of values that can change over time, %Laminarity captures how a signal is organized in terms of similar absolute values that are stable over time. Also, %Determinism—as the name hints at—is often better suited to quantify temporal structure of highly deterministic signals, while %Laminarity is often better suited to quantify temporal structure of highly stochastic signals (see also our analyses of synthetic signals above). To validate our results, we will employ both of these measures in the following analyses.

For our two sets of artificial signals, %Determinism in the case of low synchrony (Fig 1a and 1b) is 18.6% (continuous signals, Fig 2) and 7.7% (stochastic signals, Fig 3), while %Determinism in the case of high synchrony is 54.1% (continuous signals, Fig 2) and 33.6% (stochastic signals, Fig 3). Using %Laminarity, in the case of low synchrony we obtain 58.9% (continuous signals, Fig 2) and 8.8% (stochastic signals, Fig 3), compared to the cases of high synchrony, were %Laminarity is 63.5% (continuous signals, Fig 2) and 35.5% (stochastic signals, Fig 3).

For the actual hand movement acceleration and heart rate data, each individual data set was normalized by z-scoring before analysis to eliminate mere effects of signal magnitude on the estimation of synchrony. Then, the four-hand-movement acceleration time series and the two heart rate time series were embedded into a phase space in order to calculate the recurrence plot. The embedding parameters were determined based on the individual, single hand movement accelerations and heart rate profiles, using the first local minimum of the average mutual information function to estimate the delay parameter (DEL) and the first local minimum of the false-nearest neighbor function to estimate the dimensionality (DIM) of the phase-space [33]. We picked values for these parameters that fit well the overall sample for hand movements and heart rate, and used these same parameters across all data sets in order to compare them. For hand movement accelerations, we used DEL = 5 and DIM = 2 (which yields an eight-dimensional phase-space, as each pair of participants contributes 4 hand movement acceleration time series). For heart rate profiles, we used DEL = 2 and DIM = 3.

We arrived at the values for DEL and DIM employing the following steps using Norbert Marwan’s CPR toolbox for MatLab [29]: First, we started estimating the DEL parameter using the average mutual information function for each individual hand acceleration and heart rate data set. We picked the first local minimum of this function for each data set and averaged the value separately for hand movement and heart rate signals. This value was rounded up and was used for all data sets. Then, we used the false-nearest-neighbor function for each individual hand acceleration and heart rate signal to estimate the embedding dimension parameter. Again, we picked the first local minimum of this function for each data set and averaged the value separately for hand movement and heart rate signals. This value was rounded up and was used for all data sets. Note, however, that the resulting DIM-values were divided by four for hand movement acceleration and by two for heart rate, as the signals were embedded together in MdRQA. Hence, not all of the dimensions had to be reconstructed by the method of time-delayed embedding, but were available as separately measured signals (i.e., individual hand movement signals were estimated to be eight-dimensional using the false-nearest-neighbor function; however, each data set was comprised of 4 hand movement signals, yielding a four-dimensional signal. Hence, to achieve a dimensionality of eight, the 4-dimensional signals was only embedded once via time-delayed embedding, resulting in an 8-dimensional phase-space).

In both cases, the Euclidean norm was used to rescale the phase-space. A threshold of 25% of the Euclidean norm was adopted for hand movement acceleration and a threshold of 35% of the Euclidean norm was adopted for heart rate profiles.

**Fig 4:**
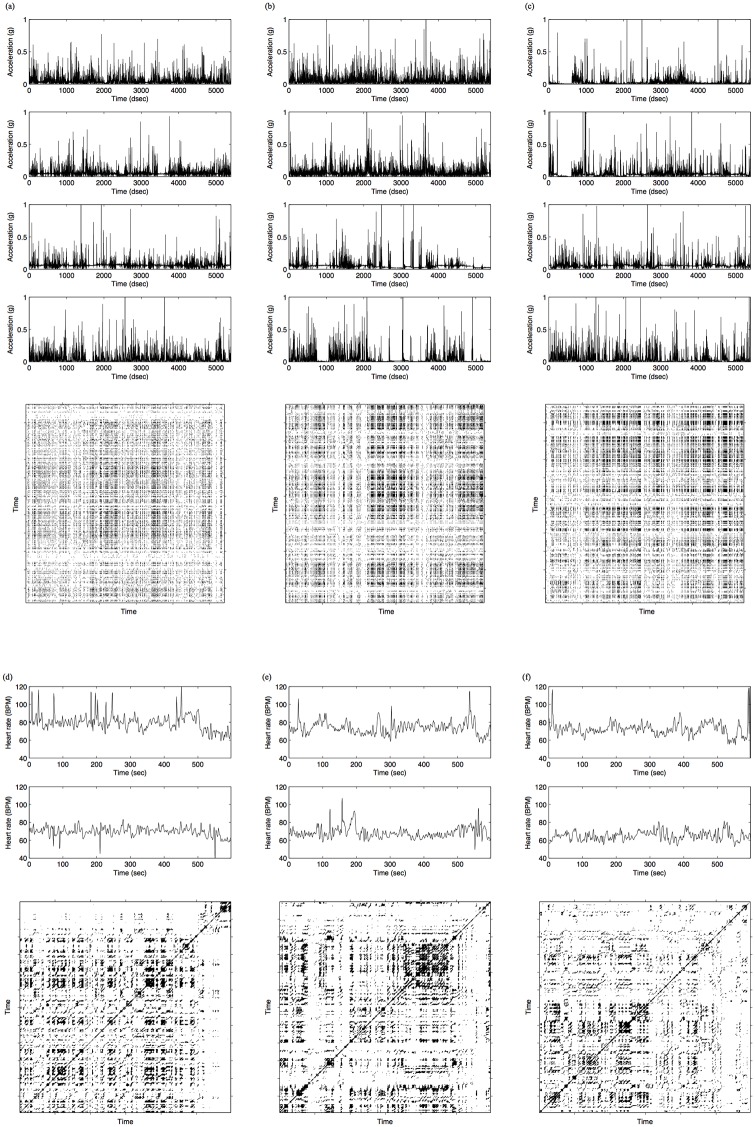
Example time series and recurrence plots from one dyad’s hand movement accelerations (top panel) and heart rates (bottom panel) during each of the three conditions: EC (left panel), HC (middle panel), and TT (right panel). (a) Hand movement accelerations and their recurrence plot during EC. (b) Hand movement accelerations and their recurrence plot during HC. (c) Hand movement accelerations and their recurrence plot during TT. The four time series displayed in (a-c) present the acceleration of participant A’s right hand, participant A’s left hand, participant B’s right hand, and participant B’s left hand, in that order from top to bottom. (d) Heart rates and their recurrence plot during EC. (e) Heart rates and their recurrence plot during HC. (f) Heart rates and their recurrence plot during TT. Please note that the recurrence plots might not present an accurate visualization, as the resolution with respect to the underlying data points is too coarse-grained.

We chose MdRQA to compute measures of synchrony because it allowed us to integrate information from multiple (i.e., more than two) time series [27], and would also capture temporal correlations between time series that do not exhibit well-defined phases (such as rocking in rocking chairs [11]), which was the case with our data—see Fig 4 for examples of hand acceleration and heart rate time series, as well as their associated recurrence plots. Moreover, bivariate scatter plots of the majority of pairs of signals did not conform to a linear (or curve-linear) relationship. Hence, linear correlation analysis was not considered. Note that RQA-based methods are powerful time-series analysis tools that also lend themselves to the quantification of attractor properties when the underlying data is recorded from deterministic systems [34]. This is not the case (and cannot readily be assumed) for our data. We are merely interested in using RQA as a correlational method that quantifies the correlational strength of a relationship between multiple signals for the reasons provided. For a detailed description of MdRQA and MATLAB code to run the analysis, see [27].

To measure the effect of synchrony due to online action coordination versus task constraints, false-pair surrogate data sets were constructed [19]; to that end, records of participants that performed the same task, but with a different partner, were randomly matched. Matching was done in the following manner: If, for example, a real pair of participants, A and B, built cars together in building condition EC, then the data of A during EC was matched with a randomly selected other participant that also performed EC. Similarly, data of B during EC was matched with another randomly selected participant that had performed EC as member of a different pair. Then, MdRQA was performed on the two false pairs, and the resulting values for %Determinism and %Laminarity were averaged, yielding a single false-pair-surrogate-value for each of the two measures, estimating the degree of synchrony when each participant of the original pair was matched with a participant that he or she did not interact with, but who performed building under the same conditions. The rationale behind the false-pair analysis is that the degree of synchrony observed within dyads that actually interacted during the building process contains synchrony due to active online coordination *and* task constraints, while any amount of synchrony observed in the false pairs must be due to task constraints alone.

The data were analyzed using linear mixed models. In the following results section, we try to present the results of the analyses in a concise and assessable manner. Due to the comparatively large number of models, presented results will be confined to a minimal set of statistics in the text, or through figures. A more extensive presentation of the different models in tabular form can be found in the Supporting Information. Before assessing the effect of the different building conditions on the dependent variables, preliminary analyses were conducted to test for potential carry-over effects, as all building conditions were recorded within-participant groups. Also, a previous analysis showed that at least one of the product outcome measures, car size, significantly increased across the four sessions [35]. Specifics of each model and analysis are presented together with the results. In general, dyad was included as a random factor in the analyses. All variables were grand-mean centered [36].

## Preliminary Analyses

### Effects of session

As our data come from a within-participants design (i.e., all dyads performed all of the three types of conditions), it is possible that learning effects will be observed on the various processes (i.e., hand movements, heart-rate) or outcome measures (i.e., self-reports, product performance measures—for example changes in number of pieces used as a function of building session number [35]), and that those effects might have a differential impact on the different building conditions. Hence, we first tested the hypothesis that session order influenced those measures by specifying a model containing the intercept, the within-dyad factors session order (4 levels: 1, 2, 3, 4), and the within-dyad factor building condition (2 levels: EC, HC, TT), as well as the interaction between the two factors. Moreover, dyad was added as a random factor.

Effects of building condition will be discussed in subsequent sections. Two of the variables—self reported effort and number of pieces used—showed a main effect of session order: participants reported significantly smaller effort ratings for the first session compared to all other sessions (*B*_*1*_ = -21.63, *t* = -3.56, *p* < .001), and dyads also used fewer pieces to build the car during the first session compared to all other sessions (*B*_*1*_ = -20.17, *t* = -7.57, *p* < .001). No other effect was apparent (all *p* > .207). These results indicate that participants needed to familiarize themselves with the building task during the first session as opposed to consistent learning effects observed throughout sessions.

As for the interaction with building condition and session number, we found no significant interactions effects for any of the measures (all *p* > .188). This means that session effects were fairly independent of effects that the building conditions had on the process and outcome measures. Hence, we can proceed with separate analyses for the effects of building conditions in the next sections.

### Comparison of the first and second hierarchical building condition

For subsequent analyses, we pooled the data from the two HC sessions that each dyad performed in order to have equal weighting for each condition type in the following analyses of building condition effect (recall that we have three different building conditions—EC, HC, and TT—but that we collected the HC session twice for each dyad so that each participant could be in the role of the chief designer). Before aggregating the data from the two HC sessions into one compound session for each dyad, we compared the average statistics for the first and second HC session that each dyad performed via dependent sample *t*-test. However, none of the process or outcome measures yielded a significant effect of first vs. second HC session (all *t* < 1.57, all *p* > .122). Hence, it seems warranted to average across the two HC sessions.

## Results

### Effects of condition on the process dynamics

To gauge the effect of the different building conditions (EC, HC, and TT) on hand movement synchrony and heart rate synchrony, we specified a model with the fixed effects intercept, building condition (EC, HC, TT), data type (real pairs, false pairs), and their interaction, and dyad as a random factor. TT is the reference category for all reported effects.

**Fig 5:**
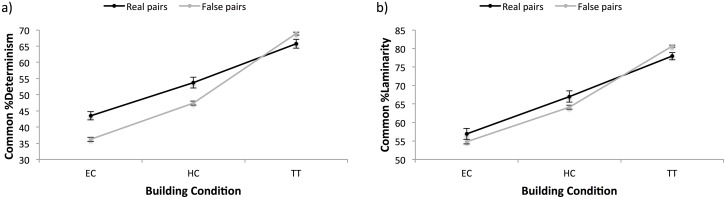
Synchrony in hand movement accelerations as a function of building condition (EC, HC, TT) and data type (real pairs, false pairs). Panel a) displays the results for %Determinism, panel b) displays the results for %Laminarity.

The analysis of the degree of hand movement synchrony between conditions, and false and real pair-data of participants revealed effects of condition and pair type: shared %Determinism in hand movements was higher in TT compared to HC (*B*_*HC*_ = -12.00, *t* = -7.93, *p* < .001), which in turn was higher compared to EC (*B*_*EC*_ = -22.27, *t* = -13.99, *p* < .001). Moreover, false pairs showed generally lower %Determinism compared to real pairs, but this effect depended on condition: false pairs yielded lower levels of shared %Determinism compared to real pairs for HC (*B*_*HC*,*false*_ = -9.44, *t* = -5.59, *p* < .001) and EC (*B*_*EC*,*false*_ = -10.36, *t* = -5.61, *p* < .001), but higher levels for TT (*B*_*TT*,*false*_ = 3.11, *t* = 2.23, *p* < .05) (Fig 5). However, we observed no significant difference for heart rate synchrony between real and false pairs, nor between the different building conditions (all *p* > .130).

Similarly, shared %Laminarity was higher in TT compared to HC (*B*_*HC*_ = -10.84, *t* = -7.83, *p* < .001), which in turn was higher compared to EC (*B*_*EC*_ = -20.93, *t* = -13.91, *p* < .001). Moreover, false pairs showed generally lower %Determinism compared to real pairs, but this effect depended on condition: false pairs yielded lower levels of shared %Determinism compared to real pairs for HC (*B*_*HC*,*false*_ = -5.61, *t* = -3.69, *p* < .001) and EC (*B*_*EC*,*false*_ = -4.78, *t* = -2.71, *p* < .01), but higher levels for TT (*B*_*TT*,*false*_ = 2.71, *t* = 2.69, *p* < .01). Just as with %Determinism, %Laminarity of heart rate did not reveal any significant effects of condition of data type, although we observe marginal effects of building condition indicating a tendency for heart rate synchrony to be lower in HC (*B*_*HC*_ = -2.47, *t* = -1.90, *p* = .068) and EC (*B*_*EC*,_ = -2.28, *t* = -1.97, *p* = .059) compared to TT.

Heart rate synchrony between real and false pairs did not differ significantly, nor did we observe an effect of building conditions on heart rate synchrony. This means, that heart rate seems to have been inherently insensitive to the social dynamics in our building task, as well as to our experimental manipulations. Hence, we discontinued further analysis of the heart rate data from here on and focus only on hand movement synchrony [37]. See the Supporting Information for a summary of the models (S4–S7 Tables).

### Effects of condition on subjective perception

To assess the effect of the different building conditions (EC, HC, and TT) on participants’ subjective perceptions, we specified a model with the fixed effects intercept and building condition (EC, HC, TT), and dyad as a random effect, separately for each of the six questions.

**Fig 6:**
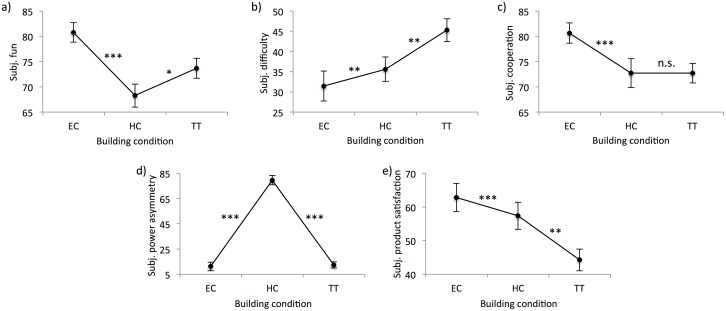
Effects of building condition (EC, HC, TT) on subjective perceptions for a) fun, b) difficulty, c) cooperation; d) power asymmetry, and e) product satisfaction. Lines marked with * denotes *p* < .05, ** denotes *p* < .01, *** denotes *p* < .001, and n.s. denotes mean differences that are not significant (i.e., *p* > .05).

The different building conditions resulted in different subjective evaluations of the building process: generally, the dyads reported more fun, better cooperation, less difficulty, and greater satisfaction with the cars for EC compared to HC, and HC compared to TT. Furthermore, dyads reported significantly greater power asymmetry for HC compared to EC and TT, validating the fact that participants assumed different roles in HC (see Fig 6 for a summary of the effects). However, building condition did not influence subjective ratings of effort. For a summary of the models, see the Supporting Information (S8 Table).

### Effects of condition on product outcomes

To gauge the effect of the different building conditions (EC, HC, and TT) on objective outcome measures, we specified a model with the fixed effects intercept and building condition (EC, HC, TT), and dyad as a random effect, separately for each of the three aspects of product quality.

**Fig 7:**
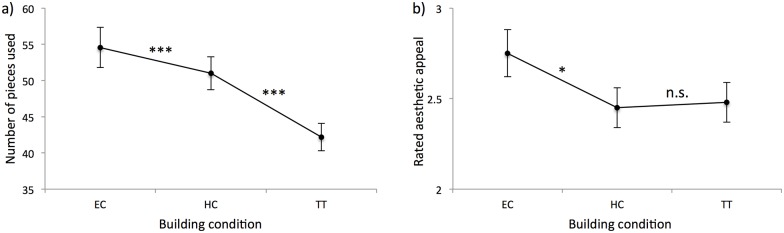
Effects of building condition (EC, HC, TT) on product outcomes for a) number of pieces used, and b) aesthetic appeal. Lines marked with * denotes *p* < .05, ** denotes *p* < .01, *** denotes *p* < .001, and n.s. denotes mean differences that are not significant (i.e., *p* > .05).

Cars built during EC and HC were composed of more pieces compared to TT. Furthermore, cars built during EC were judged to be more aesthetically appealing compared to HC and TT (see Fig 7 for a summary of the effects). However, the distance that cars traveled on the ramp test did not differ as a function of condition. The models are summarized in the Supporting Information (S9 Table)

### Relation between synchrony and subjective outcomes

To quantify the relationship between the degree of hand movement and subjective perceptions, we specified a series of models with the degree of hand movement synchrony (shared %Determinism of hand movement activity) as predictor variables, and the dyad value of the six questionnaire items as dependent variables. Furthermore, we added an interaction term with the factor building conditions (EC, HC, and TT) to examine whether the relation between the predictor and dependent variables was moderated by the different building conditions. Dyad is added as a random effect.

We observed interactions between building condition and the effect of synchrony in hand movements on perceptions of fun and cooperation: hand movement synchrony—as captured by %Determinism—was *negatively* associated with fun during EC (*B* = -56.23, *t* = -3.73, *p* < .001). For perceived cooperation, synchrony in hand movements was *negatively* associated with cooperation ratings during EC (*B* = -45.53, *t* = -3.64, *p* < .001), but *positively* associated with cooperation rating during HC (*B* = 53.07, *t* = 2.59, *p* < .05). No further effects were observed, and no effects of synchrony in hand movements were observed for TT. Furthermore, the degree of synchrony in hand movements (*B* = 39.88, *t* = 2.97, *p* < .01) was generally *positively* associated with perceived task difficulty across all conditions. No other significant effects of hand movement synchrony were observed for the other self-report items.

Also, we observed the same interactions between building condition and the effect of synchrony in hand movements on perceptions of fun and cooperation when using %Lamniarity: synchrony of hand movements (*B* = -49.47, *t* = -3.78, *p* < .001), was *negatively* associated with fun during EC. For perceived cooperation, synchrony in hand movements was *negatively* associated with cooperation ratings during EC (*B* = -28.57, *t* = -4.01, *p* < .001). Also, %Laminary revealed a positive relation between the degree of synchrony in hand movements and perceived difficulty across all three conditions (*B* = 43.19, *t* = 3.05, *p* < .01). See the Supporting Information of a summary of the models (S10 and S11 Tables).

### Relation between synchrony and product outcomes

To gauge the relationship between the degree of synchrony in hand movements and objective outcome measures, we specified a series of models with the degree of synchrony in hand movements as predictor variables, and the values of the three outcome variables as dependent variables. Again, we added an interaction term with the factor building conditions (EC, HC, and TT) to examine whether the relation between the predictor and dependent variables was moderated by the different building conditions. Dyad was added as a random effect. Using shared %Determinism as predictor, hand movement synchrony was not predictive of car performance on the ramp test. However, the degree of synchrony in hand movements was *negatively* associated with the number of pieces used (*B* = -70.26, *t* = -7.53, *p* < .001) and aesthetic appeal across all conditions (*B* = -1.42, *t* = -3.76, *p* < .001).

When using %Laminarity, synchrony in hand movements was *negatively* associated with the number of pieces used (*B* = -76.62, *t* = -7.59, *p* < .001) and aesthetic appeal across all conditions (*B* = -1.55, *t* = -4.00, *p* < .001), just as with %Determinism. Again, no effects of hand movement synchrony on car range traveled were apparent. See the Supporting Information for a summary of the models (S12 Table).

**Fig 8:**
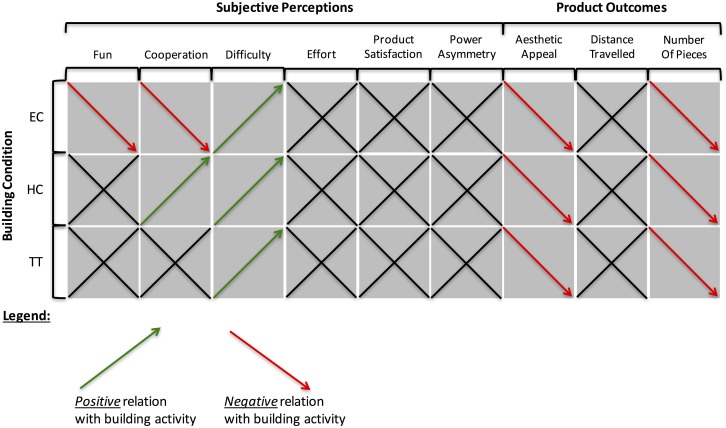
Schematic summary of the effects of synchrony in hand movement acceleration and heart rate on subjective perceptions and objective product outcomes by building condition.

Fig 6 summarizes the effects of synchrony on subjective perception and product outcomes schematically. When observing Fig 8, the following overall picture emerges: 1.) in the complex joint building task investigated here, synchrony generally shows a negative relationship with outcome variables that are related to the successful completion of the task; 2) this, however, also depends on building condition: while synchrony measures in EC show a negative relationship with positive outcome variables throughout, this is not the case for HC. In HC, product outcomes are also negatively related to synchrony (as in EC), but subjective outcomes regarding the social aspects of interaction between members of the dyad are positively related to synchrony, as reported in many previous studies. We will discuss this difference between conditions in more detail below. Finally, and as expected, synchrony measures in TT were less informative with regard to the outcome variables (i.e., fewer significant effects were observed in this condition), as online interaction was severely limited in this condition due to the experimental instructions.

## Discussion

Participants found the building process during TT more difficult and were less satisfied with their product compared to EC and HC. They also rated cooperation higher in EC compared to HC and TT, and reported more fun in EC compared to HC and TT. Regarding task outcomes, participants used more pieces for cars in EC and HC compared to TT, and cars were rated as more aesthetically pleasing in EC compared to HC and TT. Even though participants were not explicitly told to use as many pieces as possible, increasing the aesthetic appeal of cars by virtue of adding ornamental pieces can explain this effect at least in part [35]. In sum, subjective and objective outcome measures, as a function of the three conditions, suggest that freedom of interaction is not only more satisfying subjectively, but also more functional, leading to better results.

The relation between the product outcome measures and synchrony conformed to a comparatively simple pattern, where the degree of hand movement synchrony was *negatively* associated with aesthetic appeal and car size. However, no effect was found for the ramp test (i.e., the degree of hand movement synchrony did not predict how far the car would travel after being rolled down a ramp in a standardized manner). Of course, this outcome measure might simply be relatively insensitive compared to the other two. However, it might also be the case that our participants lacked—by large—a certain specialist knowledge that one would need to build a car that would have good travelling qualities (i.e., a heavy car, with a certain distribution of weight among the chassis and a certain relation between the width of the axes and the length of the chassis etc.). While most participants had a good idea of what would make a car look beautiful, judging by their course of study, most would not have known anything about engineering-aspects of a model car. And if participants did not know how to achieve this goal in the first place, changes in their coordination dynamics would neither have helped nor hurt in that regard.

The effects of hand movement synchrony on perceived difficulty were straight forward, the effect on perceived fun and cooperation were more complicated, and depended on the building conditions: Among all conditions, increased synchrony of hand movements was *positively* associated with perceptions of difficulty. However, while the degree of hand movement synchrony was *negatively* associated with fun and cooperation in EC, for HC, hand-movement synchrony was *positively* associated with reported cooperation. Thus, EC shows a consistent effect pattern where there is a negative correlation, if any, between synchrony and measures of product outcomes and positive subjective perceptions. HC, by contrast, shows a distinction between performance (product outcomes, negative correlation) and social (subjective perceptions, positive correlations) aspects. We suggest that the hierarchical relationship induced in HC might trap participants in a situation where the focus on the relationship aspect of the task favors a synchronous mode of interaction, while the focus on the production aspect favors non-synchronous mode of interaction: it feels good to synchronize with your boss, but this is not helpful for the production process. This interpretation is also supported by the generally lower quality of car models (judged by the number of pieces participants were able to use in the 10-minute period and the aesthetic appeal ratings).

Still, both EC and HC allowed many opportunities for online interaction between participants. In contrast, if participants’ opportunities for online interaction were significantly constrained (TT), measures of synchrony are not very informative about the interaction process. As the surrogate analysis of synchrony in hand movements showed, synchrony was even slightly *higher* among the false pairs compared to the real pairs. This is because the false pairs exhibit a greater probability to coincide on periods of inactivity and activity (which are otherwise fairly complementary in the real pairs, like turn taking in a conversation). This highlights how synchrony in TT is strongly driven by the task constraints (i.e., the rigid turn-taking behavior imposed by the task instruction), as opposed to online coordination during interaction, and does not show unique significant effects of synchrony on subjective measures.

In contrast, when participants can freely interact, synchrony is predictive of subjective and objective task outcomes. However, and contrary to the results of many prior studies, synchrony was overall *negatively* associated with subjective and objective outcome measures (cf. [37], showing that synchrony in electrodermal activity in teams is and marker of conflict, not cooperation) with the exception, discussed above, of the positive relation between hand movement synchrony and subjective perception of fun and cooperation in HC. This might be because in this condition, participants possibly focused not only on the achievement of an end-product, but on the coordinative process between them as well: here, synchrony was *positively* related to reported subjective ratings of fun and cooperation; that is, rapport, replicating previous findings (albeit still *negatively* related to product quality).

The absence of effects of subjective perceptions of power-asymmetry might be due to the fact that this factor only played a role in the HC condition, while the absence of effects of subjective perceptions of effort might be due to the inherent ambiguity of the question: While effort ratings were positively correlated with difficulty ratings, only difficulty was negatively correlated with fun (see S3 Table in the Supporting Information). Hence, participants might have interpreted the question of effort not in direct opposition to positive aspects of the task (i.e., one might also make an extra effort because one cares, is very motivated to perform or has a fun during the task), giving this question a more ambiguous nature. It remains unclear why there are no effects of synchrony on the perceived satisfaction with the resulting model car, especially since the satisfaction ratings of the dyads are correlated—if also only weakly—with the satisfaction ratings of the independent raters (see again S3 Table in the Supporting Information), which are associated with lower degrees of hand movement synchrony.

Nevertheless, the pattern of results observed provides an interesting extension of current work on synchrony in joint action: Firstly, synchrony might be a function of the goal of the interaction. Whereas the positive effects of synchrony have been observed in joint action studies where interpersonal coordination was the primary goal, in this study joint action was a means for achieving a product, leading to significantly different findings regarding synchrony. This is validated by the finding that when participants were asked to focus on the interaction process as well (HC), positive relations between synchrony and rapport were observed.

Moreover, it is noteworthy that the joint action studies that found effects of synchrony in more ecological settings (natural conversation/flirting [14], therapist-client-interactions [15], classroom interactions [13]) were all about the social relations/emotions between the participants involved, not about working towards an external goal or satisfying other external goal- and task-constraints (however, one could argue that therapist-client-interactions are driven by achieving an external goal as well). Hence, when the task-focus is implicitly or explicitly lying on the social dimension of the interaction, then synchrony might be a positive driving factor—or marker—for the quality of that interaction, just as we see in the differences between our EC and HC conditions. Also, effects of synchrony are more nuanced in those studies, not showing a constant synchrony effect throughout the observed periods (as in rocking in rocking chairs, for example [11]), but rather a kind of complicated [14] or intermittent [15] form of synchronous interaction.

Secondly, synchrony might be a function of task complexity. In a complex task, such as utilized in the present study, a kind of “division of labor strategy” makes sense [38]. Here, synchronization could be a disadvantage in goal attainment, as it does not provide sufficient behavioral flexibility. Rather, it might be more productive to perform complementary actions at the same time or similar actions at different times, leading to a greater diversification of action. These findings seem to align themselves with recent studies that demonstrated the importance of complementary dynamics in joint action given certain task constraints [17, 18]; further, they are in agreement with one of the eminent theories of the social sciences about the nature of cooperation, namely the theory of division of labor [39], where task productivity can be increased if participants cooperate toward task achievement not by doing similar things at similar times, but by specializing their actions to do different things at similar or different times. In line with this speculation is also a study by Abney and colleagues [40], where dyads were asked to build the “tallest possible tower” within a 15-minute-period using uncooked spaghettis and marshmallows. The researchers measured the amount of participants’ body movement during the task, and similar to our findings, participants performed better (i.e., built bigger towers) when their body movements were less synchronized during task.

The relation between the emerging concept of complementary interaction and our results (as well as the ones, for example, found by [40]) can be understood as follows: Synchronous interaction, if observed, is always a version of doing more or less the same thing at more or less the same time. If the degree of synchrony is positively associated with performance of subjective well-being, then synchrony is a “good choice” to coordinate the interaction. Now first, as we have also briefly mentioned above, the relation between synchrony and complementarity is a little bit like the relation between linearity and nonlinearity: If we find the above described condition satisfied, we can call an interaction pattern synchronous. The failure to observe synchrony, however, does not imply that action is uncoordinated. Coordination might proceed according to a more complicated, complementary pattern, with the number of such relationships being potentially infinite. Hence, unless one does not know what to look for a priori, a specific complementary pattern will be probably be missed [19]. However, general evidence for the presence of complementary interaction might come from observations of negative relations between synchrony and some performance/well-being outcome measure, as it might be difficult to achieve an increase of synchrony and complementarity at the same time. Rather, one comes at the expense of the other (at least within the same observable), and greater degrees of functional complementary interaction will degrease the degree of synchrony, and can—at the same time—establish a (negative) correlation with the degree of synchrony and some outcome measure. According to this logic, negative effects of synchrony (or positive effects weak coupling [40]) are indicative of the presence of complementary interaction, but are an underspecifying source of information with regard to what pattern of (complementary) interaction is realized specifically. In any case, one can speculate that the presence of an external task goal and/or the layout of the task structure being complex seem to favor complementary kinds of interaction, as we have discussed above.

That task structure plays a critical role in setting up coordinative dynamics, as well as the potential benefits and disadvantages of a specific coordination pattern is also highlighted by the results of the false-pair surrogate analysis, which suggests that behavioral synchrony due to task constraints increased from EC to HC to TT. However, behavioral synchrony due to participants’ interaction (i.e., the difference between synchrony observed in real and false pairs) was greater when participants were allowed to interact (EC, HC) compared to when online interaction was prohibited (TT), in line with previous findings in joint action [11]; however, this was only observed in hand movements, not in heart rate.

### Limitations of the study

The absence of a genuine synchrony effect of the heart-rate measure that we observe throughout our analyses might be because the task did not involve any significant degree of functional coupling between heart rate and task demands (e.g., through breathing) [41,42]; with regard to evoking effects of heart-rate in the current study, a limitation is surely that the task did not evoke strong emotional reactions, where heart rate dynamics might index shared emotions [6]. Alternatively, this could be due to methodological limitations, as heart rate was recorded as BPM averaged across overlapping 5-second intervals, which might have smoothed-out some of the relevant dynamics [43]. In this context it is also worth mentioning that other research investigating shared heart-rate dynamics in joint action have found that much of the subjective-emotional responses are more strongly anchored on behavioral measures of joint action, not heart-rate coordination [44]. Finally, interpersonal interaction has been shown to work on different levels and simultaneously employ multiple channels of communication [45,46]. However, our study did not investigate aspects of verbal communication or gesture, which could have mediated the effects observed in our measures, and it is certainly possible that measures related to verbal communication could have yielded different coordination dynamics than what we observed for the hand movements and heart rate. Future research would certainly benefit from an even more encompassing combination of action and communication measures.

Another methodological limitation is the usage of MdRQA in the current study. As mentioned above, the upside of MdRQA is, that it can quantify the dynamics of multiple (more than two) signals at once, providing a “group” or “systems-level” quantification that is lost when considering only pair-wise correlations. Moreover, it thus circumvents the problems of how to deal with multiple comparison when testing multiple pairings that exceed the number of available degrees-of-freedom in the data. On the downside, MdRQA does not (in its current form) allow to investigate leader-follower relations (as in CRQA or cross-correlation analysis), where one could investigate time-correlated behavior between the signals on time-lags different than 0 (For example, it could be that two participants exhibit a more turn-taking behavior, where they change leader and follower roles during the task, and that their efficient switching in roles is an important factor for the success of the interaction). Nor does it allow for an explicit nesting of the data structure (as with linear modelling), where one could pursue questions as to what parts of the observed coordination are intra-individual, and what parts are inter-individual (for example in the case of the hand-movement coordination with a participant as opposed to between participants). Here, future methodological advancements are needed in order to investigate such questions properly.

### Conclusion

In sum, the results of our study suggest that for tasks that have comparatively low task constraints and that are complex in the sense that they allow multiple ways to interact in order to successfully achieve the task outcome, optimally coordinated joint action might actually lie on a continuum between synchrony and action diversification depending on specific constraints. Our study suggests that task complexity and degrees of freedom in coordinating joint action might be the relevant dimensions that dictate whether one or the other will be the dominant strategy.

Further research should not only look at more complex joint action tasks, but also investigate variations of task demands along a continuum of complexity. This might be needed in order to integrate the disparate findings. Moreover, conceptual work needs to sharpen the concept of complementarily in joint action, and its potential relations to, or equation with, the concepts of synchrony as well as diversification of action in order to address joint action in more ecological contexts.

## Supporting Information

S1 Appendix Questionnaire. Danish and English questionnaires.
(DOCX)

S1 Table Correlations between questionnaire responses. *Note*. S1 Table summarizes the average correlations of questionnaire responses within each dyad. The majority of responses exhibited moderately-low positively correlations, except for the question of control (i.e., “Did you or your partner direct the design of the car more?”, which was r = -.856). This was driven by the HC-condition, were one participant was assigned the role of the lead-designer, and the other one the role of the assistant. Hence, we calculate the difference between—instead of the average of—these values for our analyses to reflect this kind of perceived power-asymmetry. Correlations were computed for each questionnaire response within each dyad and correlation coefficients were then averaged for each item pair (i.e., “Fun” vs. “Fun” is the average correlation of fun-ratings within dyads).
(DOCX)

S2 Table Inter-rater correlations for aesthetic appeal. *Note*. S2 Table summarizes the correlations between the 9 raters that rated the cars for aesthetic appeal. The numbers above the diagonal represent the magnitude for the Pearson-correlation coefficients among raters. The values below the diagonal represent their associated *p*-values. Significant correlations and *p*-values < .05 are printed bold.
(DOCX)

S3 Table Correlations among subjective and objective outcome measures. *Note*. S3 Table summarizes the correlations between the subjective and objective outcome measures: Q1 (“fun”), Q2 (“difficulty”), Q3 (“effort”), Q4 (“cooperation”), Diff. Q5 (“power asymmetry”), Q6 (“product satisfaction”), car range (“distance car traveled on ramp test”), car aesthetics (“aesthetic appeal of the car”), car pieces (“number of pieces build into car in a given 10-minute building session”). The numbers above the diagonal represent the magnitude for the Pearson-correlation coefficients among the variables. The values below the diagonal represent their associated *p*-values. Significant correlations and *p*-values < .05 are printed bold.
(DOCX)

S4 Table Coefficients, standard errors, *t*-values and significance level for hand movement synchrony (%Determinism). *Note*. *t*-values marked with * denote *p* < .05, ** denotes *p* < .01, and *** denotes *p* < .001.
(DOCX)

S5 Table Coefficients, standard errors, *t*-values and significance level for hand movement synchrony (%Laminarity). *Note*. *t*-values marked with * denote *p* < .05, ** denotes *p* < .01, and *** denotes *p* < .001.
(DOCX)

S6 Table Coefficients, standard errors, *t*-values and significance level for heart rate synchrony (%Determinism). *Note*. *t*-values marked with * denotes *p* < .05, ** denotes *p* < .01, and *** denotes *p* < .001.
(DOCX)

S7 Table Coefficients, standard errors, *t*-values and significance level for heart rate synchrony (%Laminarity). *Note*. *t*-values marked with * denotes *p* < .05, ** denotes *p* < .01, and *** denotes *p* < .001.
(DOCX)

S8 Table Coefficients, standard errors, *t*-values and significance level for the six items of the subjective perception questionnaire of the as a function of building condition. *Note*. *t*-values marked with * denote *p* < .05, ** denotes *p* < .01, and *** denotes *p* < .001.
(DOCX)

S9 Table Coefficients, standard errors, *t*-values and significance level for the three the product outcomes as a function of building condition. *Note*. *t*-values marked with * denote *p* < .05, ** denotes *p* < .01, and *** denotes *p* < .001.
(DOCX)

S10 Table Coefficients, standard errors, *t*-values and significance level for the effects of synchrony measures (%Determinism and %Laminarity) on subjectively perceived fun and cooperation by building condition. *Note*. We observed interactions between condition and the effect of hand and heart rate synchrony on perceptions of fun and cooperation; hence, we broke down the analyses by the factor building condition and examined the effects of hand movement synchrony separately for each building condition. %Det. = %Determinism; %Lam. = %Laminarity; *t*-values marked with * denote *p* < .05, ** denotes *p* < .01, and *** denotes *p* < .001.
(DOCX)

S11 Table Coefficients, standard errors, *t*-values and significance level for the effects of synchrony measures (%Determinism and %Laminarity) on subjectively perceived difficulty, effort, power-asymmetry, and product satisfaction. *Note*. The degrees of hand movement synchrony was generally *positively* associated with perceived task difficulty. No significant effects of hand movement synchrony was observed for the other self-report items. %Det. = %Determinism; %Lam. = %Laminarity; *t*-values marked with * denote *p* < .05, ** denotes *p* < .01, and *** denotes *p* < .001.
(DOCX)

S12 Table Coefficients, standard errors, *t*-values and significance level for the effects of synchrony on car range, car pieces, and car aesthetic appeal. *Note*. %Det. = %Determinism; %Lam. = %Laminarity; *t*-values marked with * denote *p* < .05, ** denotes *p* < .01, and *** denotes *p* < .001.
(DOCX)
