# Supplementary material for: Beyond Synchrony: Joint Action in a Complex Production Task Reveals Beneficial Effects of Decreased Interpersonal Synchrony
Source: PLoS One. 2016 Dec 20;11(12):e0168306. doi: 10.1371/journal.pone.0168306 (PMC5172585; doi:10.1371/journal.pone.0168306)
Supplement: S1 Appendix — Danish and English questionnaires. (DOCX) [file pone.0168306.s001.docx]

**S1 Appendix**

**Questionnaire.** Danish and English questionnaires.

*(Danish original)*

Tag dig venligst tid til at overveje din oplevelse af byggeopgaven et øjeblik. Læs følgende spørgsmål og besvar dem ved at sætte en enkelt streg tværs hen over svarlinjen ved hvert spørgsmål. Svarlinjen repræsenterer en glidende skala imellem de to udsagn i hver ende.

Var byggeopgaven god underholdning?

| **Overhovedet**  **ikke**  **underholdende** | __________________________________________________ | **Særdeles**  **underholdende** |
| --- | --- | --- |

Var byggeopgaven svær?

| **Overhovedet** __________________________________________________  **ikke**  **svær** |  | **Særdeles**  **svær** |
| --- | --- | --- |

Krævede byggeopgaven meget energi?

| **Overhovedet**  **ikke energikrævende** | __________________________________________________ | **Særdeles energikrævende** |
| --- | --- | --- |

Fungerede samarbejdet mellem dig og din partner godt under byggeprocessen?

| **Overhovedet**  **ikke velfungerende** | __________________________________________________ | **Særdeles velfungerende** |
| --- | --- | --- |

Hvem ledede konstruktionen af bilen mest, din samarbejdspartner eller dig selv?

| **Mig** | __________________________________________________ | **Min**  **partner** |
| --- | --- | --- |

I hvor høj grad afspejler den bil, I byggede, dine forestillinger om en god bil?

| **Overhovedet**  **ikke** | __________________________________________________ | **I meget**  **høj grad** |
| --- | --- | --- |

*(English translation)*

Please take a minute to reflect on your building experience. Read the following questions and answer them by making a single mark that crosses the line between the conditions at either end.

"How much fun was the building task?"

**No fun at all** _____________________________________________________ **A lot of fun**

"How difficult was the building task?"

**Very easy** ____________________________________________________ **Very difficult**

“How effortful was the building task?”

**Not effortful** _____________________________________________________ **Very effortful**

"How well did you and your partner cooperate during the building process?"

**Not well at all** _____________________________________________________ **Very well**

"Did you or your partner direct the design of the car more?"

**Me** _____________________________________________________ **My partner**

"How much does the car your team constructed reflect your ideas about what

a good car should be like?"

**Not at all** _____________________________________________________ **A lot**
