# Supplementary material for: Beyond Synchrony: Joint Action in a Complex Production Task Reveals Beneficial Effects of Decreased Interpersonal Synchrony
Source: PLoS One. 2016 Dec 20;11(12):e0168306. doi: 10.1371/journal.pone.0168306 (PMC5172585; doi:10.1371/journal.pone.0168306)
Supplement: S1 Table — Note. S1 Table summarizes the average correlations of questionnaire responses within each dyad. The majority of responses exhibited moderately-low positively correlations, except for the question of control (i.e., “Did you or your partner direct the design of the car more?”, which was r = -.856). This was driven by the HC-condition, were one participant was assigned the role of the lead-designer, and the other one the role of the assistant. Hence, we calculate the difference between—instead of the average of—these values for our analyses to reflect this kind of perceived power-asymmetry. Correlations were computed for each questionnaire response within each dyad and correlation coefficients were then averaged for each item pair (i.e., “Fun” vs. “Fun” is the average correlation of fun-ratings within dyads). (DOCX) [file pone.0168306.s002.docx]

**Table S1. Correlations between questionnaire responses.**

|  | Fun | Difficulty | Effort | Cooperation | Control | Satisfaction |
| --- | --- | --- | --- | --- | --- | --- |
| Fun | .313 | .083 | .117 | .403 | -.216 | .239 |
| Difficulty |  | .143 | .568 | -.163 | -.296 | -.231 |
| Effort |  |  | .086 | .034 | -.326 | .044 |
| Cooperation |  |  |  | .270 | -.133 | .421 |
| Control |  |  |  |  | -.856 | -.211 |
| Satisfaction |  |  |  |  |  | .398 |

*Note.* Table S1 summarizes the average correlations of questionnaire responses within each dyad. The majority of responses exhibited moderately-low positively correlations, except for the question of control (i.e., “Did you or your partner direct the design of the car more?”, which was *r* = -.856). This was driven by the HC-condition, were one participant was assigned the role of the lead-designer, and the other one the role of the assistant. Hence, we calculate the difference between – instead of the average of – these values for our analyses to reflect this kind of perceived power-asymmetry. Correlations were computed for each questionnaire response within each dyad and correlation coefficients were then averaged for each item pair (i.e., “Fun” vs. “Fun” is the average correlation of fun-ratings within dyads).
