# Supplementary material for: Beyond Synchrony: Joint Action in a Complex Production Task Reveals Beneficial Effects of Decreased Interpersonal Synchrony
Source: PLoS One. 2016 Dec 20;11(12):e0168306. doi: 10.1371/journal.pone.0168306 (PMC5172585; doi:10.1371/journal.pone.0168306)
Supplement: S2 Table — Note. S2 Table summarizes the correlations between the 9 raters that rated the cars for aesthetic appeal. The numbers above the diagonal represent the magnitude for the Pearson-correlation coefficients among raters. The values below the diagonal represent their associated p-values. Significant correlations and p-values < .05 are printed bold. (DOCX) [file pone.0168306.s003.docx]

**Table S2. Inter-rater correlations for aesthetic appeal.**

|  | Rater 1 | Rater 2 | Rater 3 | Rater 4 | Rater 5 | Rater 6 | Rater 7 | Rater 8 | Rater 9 |
| --- | --- | --- | --- | --- | --- | --- | --- | --- | --- |
| Rater 1 |  | -.112 | **.142** | -.015 | .067 | **.276** | **.507** | **.148** | .092 |
| Rater 2 | .056 |  | .097 | **.325** | **.400** | .111 | **.125** | **.256** | **.233** |
| Rater 3 | **.015** | .098 |  | **.217** | **.182** | **.116** | **.269** | **.310** | **.225** |
| Rater 4 | .794 | **.000** | **.000** |  | **.264** | .111 | -.036 | **.153** | **.120** |
| Rater 5 | .251 | **.000** | **.002** | **.000** |  | **.246** | **.162** | **.284** | **.225** |
| Rater 6 | **.000** | .058 | **.047** | .058 | **.000** |  | **.253** | **.164** | **.272** |
| Rater 7 | **.000** | **.032** | **.000** | .537 | **.006** | **.000** |  | **.195** | **.224** |
| Rater 8 | **.012** | **.000** | **.000** | **.009** | **.000** | **.005** | **.001** |  | **.221** |
| Rater 9 | .115 | **.000** | **.000** | **.041** | **.000** | **.000** | **.000** | **.000** |  |

*Note*. Table S2 summarizes the correlations between the 9 raters that rated the cars for aesthetic appeal. The numbers above the diagonal represent the magnitude for the Pearson-correlation coefficients among raters. The values below the diagonal represent their associated *p*-values. Significant correlations and *p*-values < .05 are printed bold.
