# Supplementary material for: Beyond Synchrony: Joint Action in a Complex Production Task Reveals Beneficial Effects of Decreased Interpersonal Synchrony
Source: PLoS One. 2016 Dec 20;11(12):e0168306. doi: 10.1371/journal.pone.0168306 (PMC5172585; doi:10.1371/journal.pone.0168306)
Supplement: S3 Table — Note. S3 Table summarizes the correlations between the subjective and objective outcome measures: Q1 (“fun”), Q2 (“difficulty”), Q3 (“effort”), Q4 (“cooperation”), Diff. Q5 (“power asymmetry”), Q6 (“product satisfaction”), car range (“distance car traveled on ramp test”), car aesthetics (“aesthetic appeal of the car”), car pieces (“number of pieces build into car in a given 10-minute building session”). The numbers above the diagonal represent the magnitude for the Pearson-correlation coefficients among the variables. The values below the diagonal represent their associated p-values. Significant correlations and p-values < .05 are printed bold. (DOCX) [file pone.0168306.s004.docx]

**Table S3. Correlations among subjective and objective outcome measures.**

|  | Q1 | Q2 | Q3 | Q4 | Diff. Q5 | Q6 | Car range | Car aesth. | Car pieces |
| --- | --- | --- | --- | --- | --- | --- | --- | --- | --- |
| Q1 |  | .090 | .183 | **.540** | **-.287** | **.276** | .069 | .179 | .008 |
| Q2 | .348 |  | **.658** | **-.251** | -.009 | **-.251** | -.136 | -.050 | **-.250** |
| Q3 | .054 | **.000** |  | -.147 | -.006 | .093 | **-.190** | -.020 | .094 |
| Q4 | **.000** | **.008** | .123 |  | -.144 | **.313** | .055 | .082 | -.014 |
| Diff. Q5 | **.002** | .924 | .952 | .131 |  | .036 | .009 | -.078 | .048 |
| Q6 | **.003** | **.008** | .331 | **.001** | .705 |  | .180 | **.214** | .151 |
| Car range | .470 | .156 | **.045** | .568 | .922 | .059 |  | **.207** | -.110 |
| Car aesth. | .060 | .602 | .837 | .391 | .414 | **.024** | **.029** |  | .050 |
| Car pieces | .933 | **.008** | .328 | .881 | .620 | .113 | .249 | .601 |  |

*Note*. Table S3 summarizes the correlations between the subjective and objective outcome measures: Q1 (“fun”), Q2 (“difficulty”), Q3 (“effort”), Q4 (“cooperation”), Diff. Q5 (“power asymmetry”), Q6 (“product satisfaction”), car range (“distance car traveled on ramp test”), car aesthetics (“aesthetic appeal of the car”), car pieces (“number of pieces build into car in a given 10-minute building session”). The numbers above the diagonal represent the magnitude for the Pearson-correlation coefficients among the variables. The values below the diagonal represent their associated *p*-values. Significant correlations and *p*-values < .05 are printed bold.
