# Supplementary material for: Beyond Synchrony: Joint Action in a Complex Production Task Reveals Beneficial Effects of Decreased Interpersonal Synchrony
Source: PLoS One. 2016 Dec 20;11(12):e0168306. doi: 10.1371/journal.pone.0168306 (PMC5172585; doi:10.1371/journal.pone.0168306)
Supplement: S4 Table — Note. t-values marked with * denote p < .05, ** denotes p < .01, and *** denotes p < .001. (DOCX) [file pone.0168306.s005.docx]

**Table S4. Coefficients, standard errors, *t*-values and significance level for hand movement synchrony (%Determinism).**

| Effect | *B* | *SE* | *t* |
| --- | --- | --- | --- |
| Intercept | 65.74 | 1.38 | 47.72*** |
| Building Condition (HC) | -12.00 | 1.51 | -7.93*** |
| Building Condition (EC) | -22.27 | 1.59 | -13.99*** |
| Data Type (false) | 3.11 | 1.40 | 2.23* |
| Building Condition:Data Type (HC, false) | -9.44 | 1.69 | -5.59*** |
| Building Condition:Data Type (EC, false) | -10.36 | 1.85 | -5.61*** |

*Note*. *t*-values marked with * denote *p* < .05, ** denotes *p* < .01, and *** denotes *p* < .001.
