# Supplementary material for: Beyond Synchrony: Joint Action in a Complex Production Task Reveals Beneficial Effects of Decreased Interpersonal Synchrony
Source: PLoS One. 2016 Dec 20;11(12):e0168306. doi: 10.1371/journal.pone.0168306 (PMC5172585; doi:10.1371/journal.pone.0168306)
Supplement: S5 Table — Note. t-values marked with * denote p < .05, ** denotes p < .01, and *** denotes p < .001. (DOCX) [file pone.0168306.s006.docx]

**Table S5. Coefficients, standard errors, *t*-values and significance level for hand movement synchrony (%Laminarity).**

| Effect | *B* | *SE* | *t* |
| --- | --- | --- | --- |
| Intercept | 77.87 | 1.00 | 77.90*** |
| Building Condition (HC) | -10.84 | 1.38 | -7.83*** |
| Building Condition (EC) | -20.93 | 1.50 | -13.91*** |
| Data Type (false) | 2.71 | 1.01 | 2.67* |
| Building Condition:Data Type (HC, false) | -5.62 | 1.52 | -3.69*** |
| Building Condition:Data Type (EC, false) | -4.78 | 1.76 | -3.69** |

*Note*. *t*-values marked with * denote *p* < .05, ** denotes *p* < .01, and *** denotes *p* < .001.
