# Supplementary material for: Beyond Synchrony: Joint Action in a Complex Production Task Reveals Beneficial Effects of Decreased Interpersonal Synchrony
Source: PLoS One. 2016 Dec 20;11(12):e0168306. doi: 10.1371/journal.pone.0168306 (PMC5172585; doi:10.1371/journal.pone.0168306)
Supplement: S6 Table — Note. t-values marked with * denotes p < .05, ** denotes p < .01, and *** denotes p < .001. (DOCX) [file pone.0168306.s007.docx]

**Table S6. Coefficients, standard errors, *t*-values and significance level for heart rate synchrony (%Determinism).**

| Effect | *B* | *SE* | *t* |
| --- | --- | --- | --- |
| Intercept | 66.34 | 2.14 | 31.05*** |
| Building Condition (HC) | -3.26 | 1.75 | -1.86 |
| Building Condition (EC) | -2.44 | 1.55 | -1.57 |
| Data Type (false) | 1.33 | 2.25 | 0.59 |
| Building Condition:Data Type (HC, false) | 0.78 | 1.93 | 0.41 |
| Building Condition:Data Type (EC, false) | -0.40 | 1.68 | -0.24 |

*Note*. *t*-values marked with * denotes *p* < .05, ** denotes *p* < .01, and *** denotes *p* < .001.
