# Supplementary material for: Beyond Synchrony: Joint Action in a Complex Production Task Reveals Beneficial Effects of Decreased Interpersonal Synchrony
Source: PLoS One. 2016 Dec 20;11(12):e0168306. doi: 10.1371/journal.pone.0168306 (PMC5172585; doi:10.1371/journal.pone.0168306)
Supplement: S7 Table — Note. t-values marked with * denotes p < .05, ** denotes p < .01, and *** denotes p < .001. (DOCX) [file pone.0168306.s008.docx]

**Table S7. Coefficients, standard errors, *t*-values and significance level for heart rate synchrony (%Laminarity).**

| Effect | *B* | *SE* | *t* |
| --- | --- | --- | --- |
| Intercept | 75.13 | 1.63 | 45.96*** |
| Building Condition (HC) | -2.47 | 1.29 | -1.90 |
| Building Condition (EC) | -2.82 | 1.43 | -1.97 |
| Data Type (false) | 1.61 | 1.73 | 0.93 |
| Building Condition:Data Type (HC, false) | 0.25 | 1.54 | 0.16 |
| Building Condition:Data Type (EC, false) | -0.75 | 1.75 | -0.43 |

*Note*. *t*-values marked with * denotes *p* < .05, ** denotes *p* < .01, and *** denotes *p* < .001.
