# Supplementary material for: Beyond Synchrony: Joint Action in a Complex Production Task Reveals Beneficial Effects of Decreased Interpersonal Synchrony
Source: PLoS One. 2016 Dec 20;11(12):e0168306. doi: 10.1371/journal.pone.0168306 (PMC5172585; doi:10.1371/journal.pone.0168306)
Supplement: S8 Table — Note. t-values marked with * denote p < .05, ** denotes p < .01, and *** denotes p < .001. (DOCX) [file pone.0168306.s009.docx]

**Table S8. Coefficients, standard errors, *t*-values and significance level for the six items of the subjective perception questionnaire of the as a function of building condition.**

| Item/Effect | *B* | *SE* | *t* |
| --- | --- | --- | --- |
| Fun |  |  |  |
| Intercept | 73.66 | 1.98 | 37.19*** |
| HC | -5.40 | 2.28 | -2.37* |
| EC | 7.11 | 1.94 | 3.68*** |
| Difficulty |  |  |  |
| Intercept | 45.29 | 2.82 | 16.04*** |
| HC | -9.68 | 3.05 | -3.17** |
| EC | -13.84 | 3.70 | -3.74** |
| Effort |  |  |  |
| Intercept | 30.53 | 2.30 | 13.29*** |
| HC | -3.09 | 2.48 | -1.24 |
| EC | -3.07 | 2.57 | -1.12 |
| Cooperation |  |  |  |
| Intercept | 72.70 | 1.93 | 37.60*** |
| HC | 0.03 | 2.88 | 0.01 |
| EC | 7.95 | 2.01 | 3.96*** |
| Control |  |  |  |
| Intercept | 12.35 | 2.41 | 5.12*** |
| HC | 67.17 | 3.64 | 18.48*** |
| EC | -1.11 | 3.39 | -0.33 |
| Product Satisfaction |  |  |  |
| Intercept | 44.27 | 3.26 | 13.57*** |
| HC | 13.10 | 4.01 | 3.27** |
| EC | 18.55 | 4.17 | 4.45*** |

*Note*. *t*-values marked with * denote *p* < .05, ** denotes *p* < .01, and *** denotes *p* < .001.
