# Supplementary material for: Beyond Synchrony: Joint Action in a Complex Production Task Reveals Beneficial Effects of Decreased Interpersonal Synchrony
Source: PLoS One. 2016 Dec 20;11(12):e0168306. doi: 10.1371/journal.pone.0168306 (PMC5172585; doi:10.1371/journal.pone.0168306)
Supplement: S9 Table — Note. t-values marked with * denote p < .05, ** denotes p < .01, and *** denotes p < .001. (DOCX) [file pone.0168306.s010.docx]

**Table S9. Coefficients, standard errors, *t*-values and significance level for the three the product outcomes as a function of building condition.**

| Item/Effect | *B* | *SE* | *t* |
| --- | --- | --- | --- |
| Pieces used |  |  |  |
| Intercept | 38.03 | 1.89 | 20.15*** |
| HC | 10.00 | 2.10 | 4.76*** |
| EC | 13.56 | 2.68 | 5.05*** |
| Aesthetic appeal |  |  |  |
| Intercept | 2.49 | 0.11 | 23.08*** |
| HC | -0.03 | 0.12 | -0.30 |
| EC | 0.29 | 0.13 | 2.26* |
| Distance traveled | | | |
| Intercept | 163.73 | 7.27 | 22.52*** |
| HC | 4.18 | 8.16 | 0.51 |
| EC | 6.53 | 7.89 | 0.41 |

*Note*. *t*-values marked with * denote *p* < .05, ** denotes *p* < .01, and *** denotes *p* < .001.
