# Supplementary material for: Beyond Synchrony: Joint Action in a Complex Production Task Reveals Beneficial Effects of Decreased Interpersonal Synchrony
Source: PLoS One. 2016 Dec 20;11(12):e0168306. doi: 10.1371/journal.pone.0168306 (PMC5172585; doi:10.1371/journal.pone.0168306)
Supplement: S10 Table — Note. We observed interactions between condition and the effect of hand and heart rate synchrony on perceptions of fun and cooperation; hence, we broke down the analyses by the factor building condition and examined the effects of hand movement synchrony separately for each building condition. %Det. = %Determinism; %Lam. = %Laminarity; t-values marked with * denote p < .05, ** denotes p < .01, and *** denotes p < .001. (DOCX) [file pone.0168306.s011.docx]

**Table S10. Coefficients, standard errors, *t*-values and significance level for the effects of synchrony measures (%Determinism and %Laminarity) on subjectively perceived fun and cooperation by building condition.**

| DV/Predictors | *B* | *SE* | *t* |
| --- | --- | --- | --- |
| Fun - EC |  |  |  |
| %Det. | -47.38 | 14.39 | -3.29** |
| %Lam. | -42.22 | 14.28 | -2.96** |
| Fun - HC |  |  |  |
| %Det. | -12.06 | 24.93 | -0.48 |
| %Lam. | -6.21 | 27.31 | -0.23 |
| Fun - TT |  |  |  |
| %Det. | -5.56 | 14.13 | -0.69 |
| %Lam. | -6.18 | 16.37 | -0.39 |
| Cooperation - EC |  |  |  |
| %Det. | -35.36 | 11.81 | -3.00* |
| %Lam. | -29.87 | 11.80 | -2.53* |
| Cooperation - HC |  |  |  |
| %Det. | 53.07 | 20.90 | 2.58* |
| %Lam. | 52.90 | 23.25 | 2.28* |
| Cooperation - TT |  |  |  |
| %Det. | -10.34 | 14.36 | -0.72 |
| %Lam. | -15.01 | 16.57 | -0.91 |

*Note*. We observed interactions between condition and the effect of hand and heart rate synchrony on perceptions of fun and cooperation; hence, we broke down the analyses by the factor building condition and examined the effects of hand movement synchrony separately for each building condition. %Det. = %Determinism; %Lam. = %Laminarity; *t*-values marked with * denote *p* < .05, ** denotes *p* < .01, and *** denotes *p* < .001.
