# Supplementary material for: Beyond Synchrony: Joint Action in a Complex Production Task Reveals Beneficial Effects of Decreased Interpersonal Synchrony
Source: PLoS One. 2016 Dec 20;11(12):e0168306. doi: 10.1371/journal.pone.0168306 (PMC5172585; doi:10.1371/journal.pone.0168306)
Supplement: S11 Table — Note. The degrees of hand movement synchrony was generally positively associated with perceived task difficulty. No significant effects of hand movement synchrony was observed for the other self-report items. %Det. = %Determinism; %Lam. = %Laminarity; t-values marked with * denote p < .05, ** denotes p < .01, and *** denotes p < .001. (DOCX) [file pone.0168306.s012.docx]

**Table S11. Coefficients, standard errors, *t*-values and significance level for the effects of synchrony measures (%Determinism and %Laminarity) on subjectively perceived difficulty, effort, power-asymmetry, and product satisfaction.**

| DV/Predictors | *B* | *SE* | *t* |
| --- | --- | --- | --- |
| Difficulty |  |  |  |
| %Det. | 39.88 | 13.41 | 2.97* |
| %Lam. | 43.19 | 14.17 | 3.05* |
| Effort |  |  |  |
| %Det. | 8.63 | 8.16 | 1.06 |
| %Lam. | 12.79 | 23.80 | 0.54 |
| Control/Power asymmetry | | | |
| %Det. | -28.09 | 22.87 | -1.23 |
| %Lam. | -24.38 | 23.34 | -1.04 |
| Product satisfaction |  |  |  |
| %Det. | 44.20 | 29.14 | 1.38 |
| %Lam. | 41.54 | 29.18 | 1.18 |

*Note*. The degrees of hand movement synchrony was generally *positively* associated with perceived task difficulty. No significant effects of hand movement synchrony was observed for the other self-report items. %Det. = %Determinism; %Lam. = %Laminarity; *t*-values marked with * denote *p* < .05, ** denotes *p* < .01, and *** denotes *p* < .001.
