# Supplementary material for: Beyond Synchrony: Joint Action in a Complex Production Task Reveals Beneficial Effects of Decreased Interpersonal Synchrony
Source: PLoS One. 2016 Dec 20;11(12):e0168306. doi: 10.1371/journal.pone.0168306 (PMC5172585; doi:10.1371/journal.pone.0168306)
Supplement: S12 Table — Note. %Det. = %Determinism; %Lam. = %Laminarity; t-values marked with * denote p < .05, ** denotes p < .01, and *** denotes p < .001. (DOCX) [file pone.0168306.s013.docx]

**Table S12. Coefficients, standard errors, *t*-values and significance level for the effects of synchrony on car range, car pieces, and car aesthetic appeal.**

| DV/Predictors | *B* | *SE* | *t* |
| --- | --- | --- | --- |
| Pieces used |  |  |  |
| %Det. | -62.68 | 5.46 | -11.48*** |
| %Lam. | -75.32 | 10.08 | -7.47*** |
| Aesthetic appeal |  |  |  |
| %Det. | -1.13 | 0.37 | -3.06** |
| %Lam. | -1.56 | 0.38 | -4.02*** |
| Distance traveled |  |  |  |
| %Det. | -6.86 | 27.05 | -0.25 |
| %Lam. | -8.66 | 26.80 | -0.32 |

*Note*. %Det. = %Determinism; %Lam. = %Laminarity; *t*-values marked with * denote *p* < .05, ** denotes *p* < .01, and *** denotes *p* < .001.
